# Type 2 diabetes-associated gut microbial composition and short chain fatty acids differ across the epidemiologic transition in African origin populations

**DOI:** 10.1080/29933935.2026.2741991

**Published:** 2026-10-07

**Authors:** Larske M. Soepnel, Julianne A. Jorgensen, Luke Hannan, Candice Choo-Kang, Asanda Mtintsilana, Gertrude Ecklu-Mensah, Jack A. Gilbert, Kweku Bedu-Addo, Terrence E. Forrester, Pascal Bovet, Bharathi Viswanathan, Estelle V. Lambert, Julia H. Goedecke, Yang Dai, Amy Luke, Brian T. Layden, Lara R. Dugas

**Affiliations:** a Division of Epidemiology and Biostatistics, School of Public Health, Faculty of Health Sciences, University of Cape Town, Cape Town, Western Cape, South Africa; b Julius Global Health, Department of Global Public Health and Bioethics, Julius Center for Health Sciences and Primary Care, University Medical Center Utrecht, The Netherlands; c Division of Endocrinology, Diabetes and Metabolism, Department of Medicine, University of Illinois Chicago, Chicago, IL, United States; d Department of Biomedical Engineering, College of Engineering and College of Medicine, University of Illinois Chicago, Chicago, IL, United States; e Public Health Sciences, Parkinson School of Health Sciences and Public Health, Loyola University Chicago, Maywood, IL, United States; f Biomedical Research Innovation Platform, South African Medical Research Council, Tygerberg, South Africa; g Department of Pediatrics and Scripps Institution of Oceanography, University of California San Diego, San Diego, CA, USA; h Department of Physiology, School of Medical Sciences, Kwame Nkrumah University of Science and Technology, Kumasi, Ghana; i Solutions for Developing Countries, University of the West Indies, Kingston, Jamaica; j University Center for Primary Care and Public Health (Unisanté), Lausanne, Switzerland; k Ministry of Health, Victoria, Republic of Seychelles; l Research Centre for Health through Physical Activity and Lifestyle (HPALS), Department of Human Biology, University of Cape Town, Cape Town, South Africa; m School of Health and Medical Sciences, Faculty of Health, Engineering & Sciences, University of Southern Queensland, Toowoomba, Australia; n Jesse Brown Veterans Affairs Medical Center, Chicago, IL, United States

**Keywords:** Type 2 diabetes, African-origin populations, gut microbiota composition, short chain fatty acids, epidemiologic transition

## Abstract

The gut microbiota and microbially produced short chain fatty acids (SCFAs) are increasingly linked to type 2 diabetes (T2D), particularly in the context of dietary transitions from fiber-rich to more ultra-processed foods. Data in African origin populations are lacking. We examined the cross-sectional relationships between the gut microbiota, SCFAs, and T2D by performing amplicon sequencing and targeted metabolomics from fecal samples and measuring fasting glucose, insulin, and C-peptide in 1505 African origin middle-aged adults from Ghana, South Africa, Jamaica, Seychelles, and the US. Microbial diversity and SCFAs were highest among Ghanaian and lowest among US participants. T2D was associated with lower microbial diversity, but not when stratifying by site. Differential abundance analysis (adjusted for site, sex, BMI, and age) identified microbes associated with T2D: *Romboutsia* was negatively associated with T2D, while positively associated taxa included *Escherichia−Shigella sp., Dielma fastidiosa, Klebsiella,* and *Bacteroides vulgatus*. Several species that were differentially abundant by T2D showed strong correlations with SCFA concentrations, although the significance varied by site. Our results suggest that associations between the gut microbiota, SCFAs, and T2D may differ by environmental, dietary, and ecological contexts. Amid the shifting global dietary patterns, further research is needed to inform gut microbiota-targeted approaches to address the global T2D burden.

## Introduction

Type-2 diabetes mellitus (T2D) is a major public health threat, with large and increasing morbidity, mortality, and healthcare costs [1]. Globally, 828 million adults are living with diabetes, with T2D accounting for more than 90% of all cases. This number increased from 630 million cases in 1990; with the largest share observed in low- and middle-income countries (LMICs) [1]. While the etiology of T2D is complex, obesity, insulin resistance, and impaired beta cell function are key features [2,3].

The composition and function of the gut microbiota have been found to differ between individuals with and without T2D and are associated with insulin resistance and features of impaired glucose metabolism [4–6]. In addition, recent studies including individuals with metabolic diseases, obesity, and/or T2D have found that fecal transplantation from healthy donors improves insulin resistance and markers of metabolic health in the short term, [7] although contradictory findings have also been reported [8]. It is also notable that much of the existing evidence comes from European and East Asian populations [4,6–8]. Therefore, while dietary-related characteristics of the gut microbiota may present new avenues for T2D prevention and control, both the pathophysiological role of the microbiome and potential therapeutic intervention strategies remain largely underexplored in African origin populations.

One of the important roles of gut microbes is to ferment dietary fiber into nutrients, primarily short chain fatty acids (SCFAs). SCFAs, including acetate, butyrate, valerate, and propionate, are a key energy source for the intestinal epithelium and liver [9]. In recent years, SCFAs, in addition to serving as a nutrient source, [10–15] have been increasingly studied as signaling molecules key to hepatic gluconeogenesis and lipogenesis, [16,17] insulin sensitivity and secretion, gut barrier function, [10,18] and immune responses [19,20]. Both human and animal studies have suggested that SCFAs play a protective role in metabolic diseases; higher levels are associated with reduced body weight, decreased inflammation, and improved insulin sensitivity [21–23]. Butyrate has been found to correlate with an improved insulin response in oral glucose tolerance tests [15,21]. However, these studies rarely included African-origin populations. Moreover, the loss of dietary fiber, both in urbanized countries and in response to the nutrition transition in many countries in sub-Saharan Africa, may have a detrimental effect on microbially produced SCFA production, thereby increasing T2D risk.

To explore these relationships, we used data from five diverse African-origin populations from the Modeling the Epidemiologic Transition Study (METS) and the METS-Microbiome study [24,25]. The participants included 1505 male and female adults (35–55 y) of African origin from countries at various stages of development (based on the Human Development Index), [26,27] namely, Ghana, South Africa, Jamaica, Seychelles, and the United States (US). Unlike our prior METS-Microbiome study, which was primarily centered on obesity-related metabolic disturbances within this same cohort, [24,28] the objective of this study was to characterize cross-sectional associations between the gut microbiota composition and diversity, fecal SCFAs, and T2D and markers of glucose metabolism across different geographic sites. Overall, we hypothesize that gut microbiota diversity will decrease along the epidemiologic transition, with fewer fecal SCFAs and more dysbiosis associated with T2D.

## Methods

### Study population and design

The METS study originally recruited 2500 middle-aged adults (53.4% women) of African origin in 2010 from five countries, Ghana, South Africa, Jamaica, Seychelles and the US (N = 500 per site), with countries chosen to represent different stages of the epidemiologic transition [25–27]. Participant eligibility and selection have previously been described in detail [25]. The Ghanaian site was situated in a rural town of Nkwantakese; in South Africa, the study site was in Khayelitsha, the third-largest urban township in South Africa, which is a historically disadvantaged area adjacent to Cape Town; the Jamaican site was in Kingston, the capital and largest city of Jamaica; in Seychelles, the site was on the main island (Mahé); and, lastly; the US site was in Maywood, IL, a predominantly African American working-class community adjacent to Chicago.

Between 2017 and 2019, the METS cohort was extended through the METS-Microbiome study to explore the relationship between adiposity and the gut microbiota in the same METS participants. The participants provided fecal and serum samples, gut microbiota sequencing and clinical lab measurements, respectively [24]. More specifically, samples were analyzed for glucose metabolism markers (fasting insulin, glucose, and C-peptide levels), metabolic parameters (blood lipids), and inflammation (high-sensitivity C-reactive protein, hsCRP). Detailed descriptions of the METS and METS microbiome protocols for data collection, measurement, sample size, and laboratory procedures have been previously published [24,25].

Both METS and METS-Microbiome studies were approved by the Institutional Review Board of Loyola University Chicago, IL, US, which is the METS study coordinating center (LU200038; LU209537); and by the relevant institutional ethics committees at each participating site, as previously described [24,25]. All the study procedures were explained to the participants in their local languages, and the participants were provided written informed consent after being given the opportunity to ask any questions and were compensated for their participation.

### Anthropometric and biochemical measures

The participants were asked to fast at least 8–10 h prior to visiting local research clinics, where trained staff collected biological samples, performed anthropometric measurements and administered questionnaires. All study procedures were standardized across the research sites, which used the same model equipment. The participants wore light clothing and no shoes for weight (kg) and height (m) measurements, which were used to calculate BMI as kg/m^2^. Blood pressure (BP) was measured in triplicate at two timepoints at least one hour apart using an automatic digital monitor (model HEM-747Ic, Omron Healthcare, Bannockburn, IL, USA). Hypertension was defined as the mean systolic/diastolic BP > 130/80 mmHg or on current treatment, as per American Heart Association Guidelines [29].

Fasting glucose, fasting insulin, lipids (total cholesterol, LDL-cholesterol, HDL-cholesterol, and triglycerides), C-peptide, and hsCRP concentrations were analyzed from serum from fasted blood samples drawn from the antecubital fossa, as previously described [24,25,30]. T2D was determined using the American Diabetes Association diagnostic criteria defined as fasting glucose greater than or equal to 126 mg/dL (7.0 mmol/l) or by self-report of current treatment [31]. Secondary measures of glucose metabolism included fasting insulin clearance (C-peptide:insulin ratio) and homeostatic model assessments of beta(β)-cell function (HOMA-β) and insulin resistance (HOMA-IR). HOMA-β_c-pep_ (hereafter referred to as HOMA-β) was calculated using 0.27 * C-peptide [ng/mL]/fasting glucose [mg/dL] – 3.5); HOMA-IR_ins_ (hereafter referred to as HOMA-IR) was calculated as follows: fasting glucose [nmolL] *  fasting insulin [UmL])/22.5 [32–35]. Lastly, simple disposition index was calculated using a structural parameter inference approach (SPINA), a validated model‐based static function test of insulin‐glucose homeostasis calculated from modelled secretary capacity of beta cells and insulin receptor gain [36].

### Fecal short-chain fatty acid (SCFA) quantification

Fecal SCFA extraction and quantification [24,25,28,37] were conducted at the University of Illinois-Chicago Mass Spectrometry Core, as described previously [24,25,28,37]. SCFAs were extracted from 1 g of stool in 10 mL of 50% aqueous acetonitrile. Stable isotope-labeled SCFA derivatives generated using ^13^C_6_-labeled NPH were used as internal standards. The samples were processed for LC/MS-MS on an AB Sciex Qtrap 5500 coupled to the Agilent UPLC/HPLC system–Agilent Poroshell 120 EC-C18 Column (100 Å, 2.7 μm, 2.1 mm × 100 mm) coupled to an Agilent UPLC system (flow rate: 400 μl/min). The neat limit of detection for each short chain fatty acid was as follows: acetic acid: 10 nM; propionic acid: 5.3 nM; butyric acid: 3.2 nM; and isovaleric acid: 1.6 nM. SCFAs (μg/g) were presented as butyrate, propionate, acetate, and valerate individually, as well as total SCFAs (sum of 4) [24,28].

### Microbiome sequencing and data processing

The participants provided an early morning fecal sample, using a standard collection kit (EasySampler Fecal Collection Kit, Alpco, NH), which was immediately stored upon receipt at −80 °C. Fecal samples were shipped on dry ice to the Microbiome Core at the University of California San Diego (UCSD) for 16S rRNA gene processing. DNA was extracted using 96-well MagAttract Power Microbiome extraction plates, with blank controls and ZymoBIOMICS mock controls per plate (Cat. No. D6300). 16S rRNA gene sequencing was performed using the V4 hypervariable region amplified from extracted DNA with 515F-806R region-specific primers (515 F: 5′GTGYCAGCMGCCGCGGTAA3′; 806R: 5′GGACTACNVGGGTWTCTAAT3′) according to the Earth Microbiome Project [38]. Neither human DNA depletion nor microbial or viral DNA enrichment was done. Purified amplicon libraries were sequenced on the Illumina NovaSeq platform to produce 150 bp forward and reverse reads (IGM Genomics Center at UCSD) [24,28].

Raw sequencing data processed on the QIITA platform were demultiplexed, quality-filtered, and trimmed, with erroneous sequences removed. Amplicon sequence variants (ASVs) were identified using Deblur, resulting in 463,258,036 reads, 154,952 ASVs. ASV taxonomic assignment was performed using the DADA2 functions *assignTaxonomy* and *addSpecies*. ASVs were assigned taxonomy from domain to genus using the naïve Bayesian classifier, and species assignment was done by exact matching, requiring a full-length, 100% identity match of the ASV sequence to a single species in the SILVA v138.2 species reference database [39]. ASVs matching more than one species, or none, were reported at the genus level [39]. For downstream analysis, the count table, taxonomy, and sample metadata were integrated into a phyloseq object in R (R Foundation for Statistical Computing, Vienna, Austria). To ensure data quality, ASVs unassigned at the phylum level, reads aligned to non-bacterial sequences, and chloroplast and mitochondrial sequences were removed. Finally, the phyloseq object was filtered to retain features with more than ten reads and samples with more than 6000 sequences.

### Statistical analysis

Statistical analysis and data visualization was performed in R version 4.4.1 and STATA version 18 with available packages. Continuous variables are presented as median and interquartile range. Categorical variables are presented as proportions with *N* (%). To test whether participant characteristics were significantly different by site, *p*-values are two-sided and calculated with the Kruskal‒Wallis test for continuous variables and Pearson's chi-squared for categorical (*α* = 0.05). Post hoc pairwise analysis was performed using Dunn's test with Holm's correction (continuous) or two-sample tests of proportions with Bonferroni correction (categorical).

### Diversity and differential abundance

Shannon diversity index and Chao1 were calculated for all samples in R. Shannon and Chao1 were reported by site, T2D status, and T2D groups within each site, and significant differences between groups were calculated with the Kruskal‒Wallis test. Quantile regression models were additionally used to examine associations between the median Shannon index and i) site, adjusting for sex, age and BMI, and ii) T2D status, adjusted for site, sex, age, and BMI. Subsequently, the association between Shannon index and glucose metabolism markers (fasting insulin, fasting glucose, HOMA-IR, HOMA-β, and fasting insulin clearance) was assessed using linear regression models, adjusting for site, sex, age and BMI.

Beta diversity was calculated in Phyloseq with the Bray‒Curtis dissimilarity metric in R. Permutational analysis of variance (PERMANOVA) tests with 999 permutations and multiple groups correction using the vegan package in R were used to test beta diversity by T2D status and by site, adjusting for sex, age, and BMI.

For differential abundances, an additional filtering step was conducted to limit exceptionally rare taxa by removing ASVs present in <2% of the samples across all samples. To measure the multivariable associations of clinical features, metabolic metadata, and microbial taxa, we used microbiome multivariate association with linear models (MaAsLin3) [40]. The first model assessed the individual associations of each taxon with T2D status, adjusting for BMI, sex, age, and site. In a subsequent model, we additionally included SCFAs (propionate, acetate, valerate, butyrate) in the model. For all the models, the covariates were chosen *a priori* since sex, age, and BMI contribute to T2D prevalence [41]. Additionally, we know that the gut microbiota and T2D prevalence differ greatly among METS research sites [42]. Significance was assessed for both MaAsLin3 models using q < 0.05, and the significant associations were plotted. Spearman correlations, stratified by site and by diabetes group, were performed between concentrations of SCFAs and differentially abundant taxa.

### Predicted metabolic gene pathway

The metabolic gene pathways, or functional potential, were assessed with Phylogenetic Investigation of Communities by Reconstruction of Unobserved States 2 (PICRUSt2) [43]. KEGG orthologs were mapped to KEGG pathways and classes and were annotated. Subsequently, differential abundance was compared by T2D status. The relative abundance of all taxa with KOs along the KEGG pathways were summed and plotted. Log2 fold change in the predicted metabolic pathway levels was calculated between those with T2D and those without T2D, and averaged. KEGG pathways with adjusted *p*-values under 1*10^−45^ were included.

### Predictive modeling with machine learning models

To explore the contribution of microbial taxa and SCFA levels for predicting current T2D status relative to clinical factors traditionally associated with T2D risk, we used machine learning predictive models. Elastic net and XGBoost were employed for machine learning predictive models with cross-validation. The samples were randomly split into 75% for model training and 25% for model testing using care to preserve the overall distribution of the data by site in the training and testing datasets. The models were optimized with an elastic net model with the GLMnet and XGBoost machine learning model with caret in R [44,45]. In XGboost with 10-fold cross-validation and 100 repetitions, the predictive model was used to classify individuals according to T2D status. To assess the contribution of microbial taxa and SCFAs to predicting current T2D status in the model, we included these in addition to clinical measures of metabolic health (insulin, glucose, HOMA-IR, HOMA-β, fasting insulin clearance (C-peptide:insulin ratio), BMI, C-peptide, simple disposition index), blood pressure (high blood pressure, hypertension, systolic and diastolic blood pressure), lipids (total cholesterol, HDL-cholesterol, LDL-cholesterol, triglycerides), inflammation (hsCRP), site, age, and sex. A secondary model included only microbial taxa. Class imbalance was handled as part of model training by sample weighting by trainControl with caret in R following previous recommendations [46]. After 1000 boosting rounds, the model was evaluated with an area under the receiving operator characteristic curve (AUC or AUROC), precision, recall, and F1 scores. The best final model was selected using the trained hyperparameters and subsequently applied to the testing dataset. This model was then processed using Shapley additive explanations (SHAP) to assign modeling importance to each feature and impact the overall model prediction, thus enabling a better understanding of the individual predictive capacity of each variable in the model [47]. We presented the top 30 features contributing to model predictions.

## Results

Data from 1505 participants with complete biochemical and microbiota sequencing data were used in this analysis (Ghana: N = 339, South Africa: N = 303, Jamaica: N = 316, Seychelles: N = 335, and the US: N = 212). Of these, 1446 participants also had fecal SCFA quantified. Table 1 summarizes the overall participant characteristics, including clinical biomarkers. The median age was 43 y (63% women; see Table 1); the median age among the South African participants was lower than participants at the other sites (35 y). The obesity prevalence was lowest among the Ghanaian participants (27%, median BMI: 26 kg/m^2^) and highest among the US participants (63%, median BMI: 33 kg/m^2^), and the difference in BMI status between all five sites, except for Jamaica and Seychelles, was statistically significant.

**Table 1.** Descriptive participant characteristics.

	Ghana (*N* = 339)	South Africa (*N* = 303)	Jamaica (*N* = 316)	Seychelles (*N* = 335)	US (*N* = 212)	Overall (*N* = 1505)	*p*-value
Age (y)	43 [36, 49]^a^	35 [29, 41]^b^	46 [39, 51]^c,d^	45 [40, 49]^c^	47 [42, 51]^d^	43 [37, 49]	<0.001
**Sex, *n* (%)**							
Men	112 (33 %)	127 (42 %)	100 (32 %)	136 (41 %)	76 (36 %)	551 (37 %)	0.0468
Women	227 (67 %)	176 (58 %)	216 (68 %)	199 (59 %)	136 (64 %)	954 (63 %)	
BMI (kg/m^2^)	26 [22, 30]^a^	26 [22, 34]^a^	30 [25, 34]^b^	29 [25, 33]^b^	33 [27, 38]^c^	28 [24, 34]	<0.001
**Obesity, *n* (%)**							
Underweight	11 (3 %)^a^	20 (7 %)^b^	7 (2 %)^c^	2 (1 %)^c^	3 (1 %)^d^	43 (3 %)	<0.001
Normal	140 (41 %)	116 (38 %)	73 (23 %)	82 (24 %)	27 (13 %)	438 (29 %)	
Overweight	98 (29 %)	55 (18 %)	83 (26 %)	115 (34 %)	49 (23 %)	400 (27 %)	
Obese	90 (27 %)	112 (37 %)	153 (48 %)	136 (41 %)	133 (63 %)	624 (41 %)	
**Hypertension, *n* (%)**							
Hypertension	89 (26 %)^a^	124 (41 %)^b^	178 (56 %)^c^	192 (57 %)^c^	150 (71 %)^d^	733 (49 %)	<0.001
Non-hypertension	250 (74 %)	179 (59 %)	138 (44 %)	143 (43 %)	62 (29 %)	772 (51 %)	
Cholesterol (mmol/L)	5.3 [4.5, 6.0]^a,c^	4.3 [3.8, 5.0]^b^	5.2 [4.5, 6.0]^a^	5.4 [4.7, 6.2]^c^	4.9 [4.2, 5.5]^d^	5.0 [4.3, 5.8]	<0.001
LDL-C (mmol/L)	3.6 [2.9, 4.2]^a^	2.6 [2.0, 3.2]^b^	3.4 [2.9, 4.1]^a^	3.6 [3.0, 4.3]^a^	3.0 [2.5, 3.6]^c^	3.2 [2.6, 3.9]	<0.001
HDL-C (mmol/L)	1.2 [1.0, 1.4]^a^	1.2 [1.0, 1.6]^a,b,c^	1.2 [1.0, 1.4]^a,b^	1.3 [1.1, 1.5]^b,c^	1.3 [1.1, 1.5]^c^	1.2 [1.0, 1.5]	<0.001
Triglycerides (mmol/L)	0.90 [0.70, 1.3]^a^	0.80 [0.60, 1.1]^b^	0.90 [0.70, 1.3]^a^	1.0 [0.70, 1.4]^a^	0.90 [0.70, 1.3]^a, b^	0.90 [0.70, 1.3]	0.007
hsCRP (mg/L)	2.0 [1.0, 4.0]^a^	3.0 [1.0, 8.0] ^b,c^	3.0 [1.0, 7.0] ^b,c^	3.0 [1.0, 6.0] ^b^	4.0 [2.0, 9.0]^c^	3.0 [1.0, 7.0]	<0.001
Fasting glucose (mg/dL)	81 [77, 86]^a^	81 [77, 88]^a^	79 [72, 86]^b^	83 [76, 94]^a^	94 [86, 100]^c^	83 [77, 90]	<0.001
Fasting insulin (lU/L)	2.6 [1.1, 5.1]^a^	5.2 [3.2, 9.3]^b^	4.6 [2.1, 7.9]^c^	7.4 [4.7, 12]^d^	7.6 [4.6, 12]^d^	5.2 [2.7, 9.1]	<0.001
C-peptide (pmol/l)	470 [330, 620]^a^	560 [390, 700]^b^	590 [410, 770]^b^	590 [430, 760]^b^	740 [520, 990]^c^	570 [400, 760]	<0.001
HOMA-IR	0.51 [0.23, 1.1]^a^	1.0 [0.64, 1.9]^b^	0.90 [0.38, 1.7]^c^	1.6 [0.93, 2.6]^d^	1.9 [1.0, 3.3]^d^	1.1 [0.55, 2.0]	<0.001
HOMA-β	1.6 [1.2, 2.1]^a^	1.9 [1.3, 2.4]^b^	2.0 [1.4, 2.7]^c^	1.9 [1.4, 2.5]^d^	2.1 [1.5, 2.9]^d^	1.9 [1.3, 2.5]	<0.001
Fasting insulin clearance	151 [103.1, 304.7]^a^	97.8 [72.5, 130.2]^b^	111.8 [85.4, 188.6]^c^	76.8 [62.7, 101.6]^d^	98.5 [72.1, 121.1]^b^	103.2 [75.3, 150.7]	<0.001
**T2D status, *n* (%)**							
T2D	11 (3 %)^a^	7 (2 %)^a^	30 (9 %)^b^	30 (9 %)^b^	43 (20 %)^d^	121 (8 %)	<0.001
No T2D	328 (97 %)	296 (98 %)	286 (91 %)	305 (91 %)	169 (80 %)	1384 (92 %)	
Short chain fatty acids (SCFAs)	*N* = 338	*N* = 303	*N* = 291	*N* = 303	*N* = 211	*N* = 1446	
Acetate (μg/g)	4280 [3410, 5090]^a^	1240 [930, 1560]^b^	1970 [1360, 2700]^c^	3200 [2110, 4310]^d^	1450 [980, 2110]^e^	2190 [1300, 3770]	<0.001
Butyrate (μg/g)	1420 [950, 1880]^a^	770 [530, 1130]^b^	650 [400, 1030]^c^	540 [340, 810]^d^	790 [520, 1220]^b^	790 [480, 1290]	<0.001
Propionate (μg/g)	1850 [1350, 2730]^a^	890 [560, 1400]^b^	890 [620, 1310]^b^	1160 [780, 1690]^c^	840 [560, 1240]^b^	1110 [700, 1740]	<0.001
Valerate (μg/g)	90 [50, 160]^a^	120 [70, 190]^b^	110 [60, 170]^a,b^	80 [40, 130]^c^	130 [80, 190]^b^	110 [60, 170]	<0.001
Total SCFAs (μg/g)	8090 [6170, 9660]^a^	3170 [2220, 4230]^b^	3780 [2620, 5060]^c^	5140 [3660, 6930]^d^	3460 [2290, 4860]^b,c^	4510 [2900, 6740]	<0.001

Note: Values provided as median and interquartile range or number and percentage; *p*-value calculated with Kruskal–Wallis test for continuous variables and Chi^2^ test for categorical variables. Post hoc analysis using Dunn's test with Holm correction (continuous) or two-sample tests of proportions with Bonferroni correction (categorical): values sharing superscript letter do not differ significantly.

Abbreviations: BMI: body mass index; T2D: Type-2 diabetes; LDL-C: low-density lipoprotein cholesterol; HDL-C: high-density lipoprotein cholesterol; hsCRP: high-sensitivity C-reactive protein.

### Measures of T2D prevalence and glucose metabolism markers are different across the epidemiologic transition

The overall T2D prevalence was 8%, with the highest prevalence at the US site (20%) and the lowest at the South African (2%) and Ghanaian (3%) sites (*p*-value < 0.001; see Table 1). Participants from the US had a less favorable glucose metabolism profile than those at the other study sites, reflected in the highest levels of fasting glucose (94 mg/dL), insulin (7.6 IU/L), and HOMA-IR (1.9). These measures were lower at the other sites, particularly in Ghana (81 mg/dL, 2.6 IU/L, and 0.51, respectively). C-peptide and HOMA-β were similarly lowest among the Ghanaians and highest among the US participants (*p*-value across sites < 0.001), potentially reflecting an increased beta-cell insulin secretion in response to site-specific characteristics, including impaired glucose metabolism, insulin resistance, and high obesity prevalence.

### Short-chain fatty acid (SCFA) concentrations vary by site

SCFA levels were highest among the Ghanaians (median total SCFA levels = 8090 IQR 6170–9660 μg/g) and lowest in US participants (median total SCFA levels = 3460, IQR 2290–4860 μg/g) (*p* < 0.001). For individual SCFA levels, acetate was the highest across sites (median = 2190 μg/g), followed by propionate (median = 1110 μg/g), butyrate (median = 790 μg/g), and valerate (median = 110 μg/g) (Table 1). Ghanaians also had the highest individual SCFA levels except for valerate. The lowest individual SCFA levels varied by site: acetate and propionate were lowest in South African and US participants, while butyrate and valerate were lowest among participants in Seychelles.

### Microbial community diversity was dissimilar between sites and by T2D, but not when stratified by individual site

Figure 1A–E present the comparisons and associations of alpha and beta diversity indices by site, T2D, and secondary glucose metabolism markers. As expected, the alpha diversity indices were different by site (Shannon diversity *p* < 0.001; Chao1 *i* < 0.001 across sites, Figure 1A); when compared to Ghanaians, the median Shannon index was lower in participants from each of the other sites. Exploring alpha diversity by T2D in all the sites combined, the participants with T2D had a lower median Shannon index (*β* = –0.15; 95% CI: –0.26, –0.04; *p* = 0.009) compared to those without T2D (Figure 1B and C). However, no significant differences in Shannon or Chao1 diversity indices were found by T2D status within the individual sites (Supplementary Figure 1), and no significant association between Shannon diversity and T2D was found when adjusting for covariates (site, sex, age, BMI) in the quantile regression model. However, in the adjusted regression analysis, HOMA-IR and HOMA-β were inversely associated with the Shannon diversity index (*p* < 0.05), (Figure 1D).

**Figure 1.**
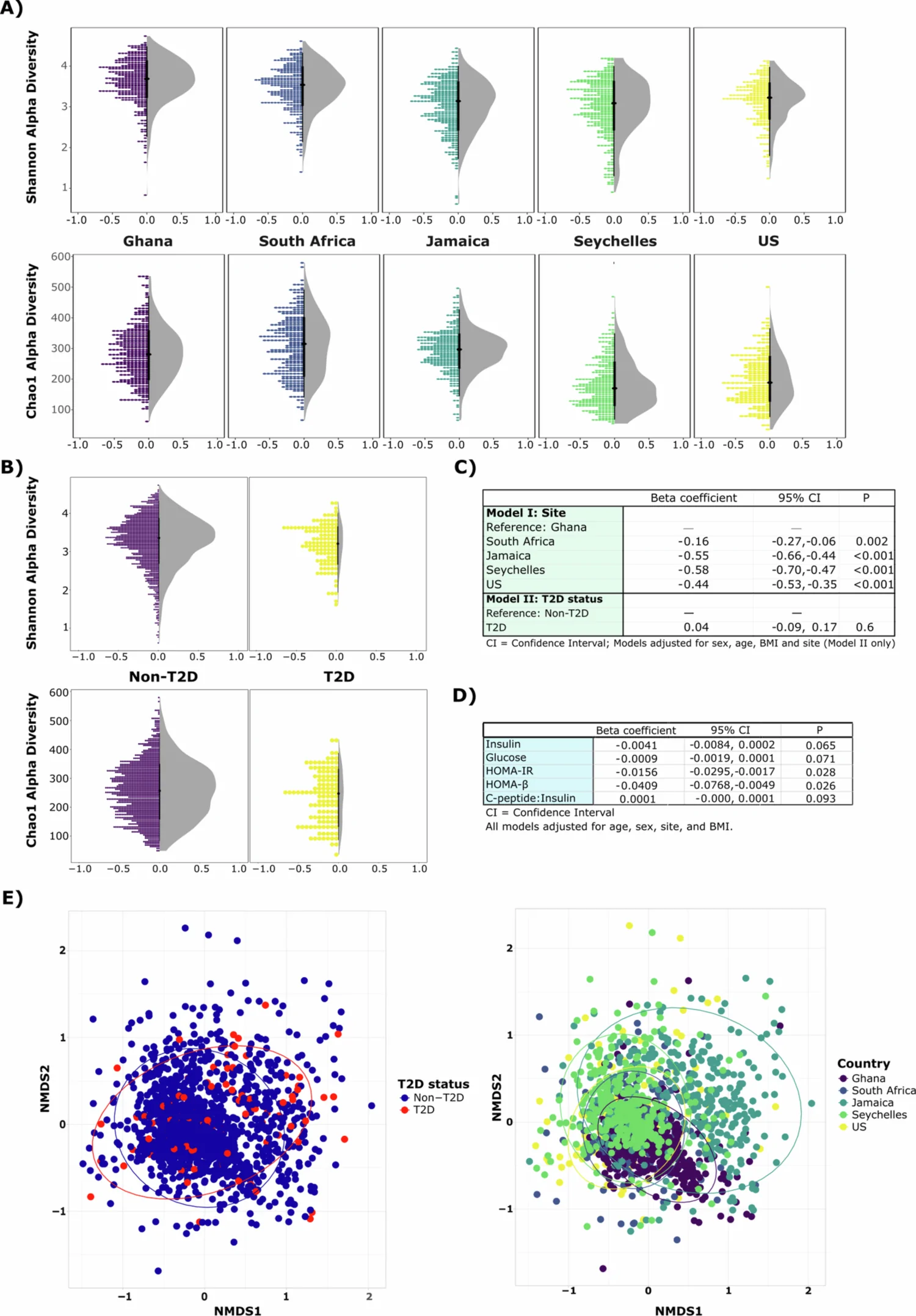
Alpha and beta diversity by site, glycemic traits and T2D status (*n* = 1505). (A) Shannon and Chao1 Alpha diversity in each site; (B) Shannon and Chao1 Alpha diversity by T2D status when pooling the sites; (C) Quantile regression models of median Shannon Alpha diversity by site (model I) with Ghana as the reference, and T2D status (model II) with non-T2D as the reference; (D) Results of five linear regression models assessing the association between Shannon Alpha diversity and individual glucose metabolism markers (fasting insulin, fasting glucose, HOMA-IR, HOMA-β_c-peptide_, and fasting insulin clearance (C-peptide:insulin ratio), with each model adjusted for age, sex, and site. (E) Beta diversity by T2D status and country of origin using Bray–Curtis dissimilarity statistic. Beta diversity statistic was significantly different by T2D status and by site, using PERMANOVA (*p* < 0.001).

For beta diversity, assessed by the Bray‒Curtis dissimilarity measure, significant differences in microbiome community composition were found between sites (Figure 1E **;** PERMANOVA *R* ^2^ = 0.092 *p* < 0.001), and by T2D status in the models adjusted for site, sex, BMI, and age (PERMANOVA *R* ^2^ = 0.098 *p* < 0.001).

### Microbial taxa differ by T2D across sites and differentially correlate with SCFA levels

Figure 2A presents the taxa that were significantly associated with T2D, as determined with a MaAsLin3 model adjusted for age, sex, site, and BMI. The abundance of multiple taxa was positively associated with T2D, including *Escherichia Shigella sp., Coprobacillus cateniformis, Dielma fastidiosa*, *Enterobacteriaceae Klebsiella,* and *Bacteriodes vulgatus.* In contrast, *Romboutsia sp.* was negatively associated with T2D. In this model, site accounted for most of the variation in the abundance of microbial taxa (Supplementary Figure 2).

In Figure 2B, butyrate, acetate, propionate, and valerate were added to the MaAslin3 model to determine their associations with gut microbial taxa, in addition to T2D (Figure 2B). In this model, many of the top taxa associated with SCFAs were from the *Lachnospiraceae* and *Prevotellaceae* families. Strong positive associations with SCFAs were found for abundance of *Prevotellaceae* (acetate and propionate), *Family XIII AD 3011 group* (valerate), *Lactobacillaceae Weissella cibaria* (acetate), and *Ruminococcaceae Gemmiger* (butyrate), among others. Many taxa had mixed associations with each SCFAs, such as *Prevotellaceae* (positively associated with acetate and propionate, negatively with butyrate) and *Lachnospiraceae Agathobacter* (negatively associated with acetate and valerate, positively with butyrate).

**Figure 2.**
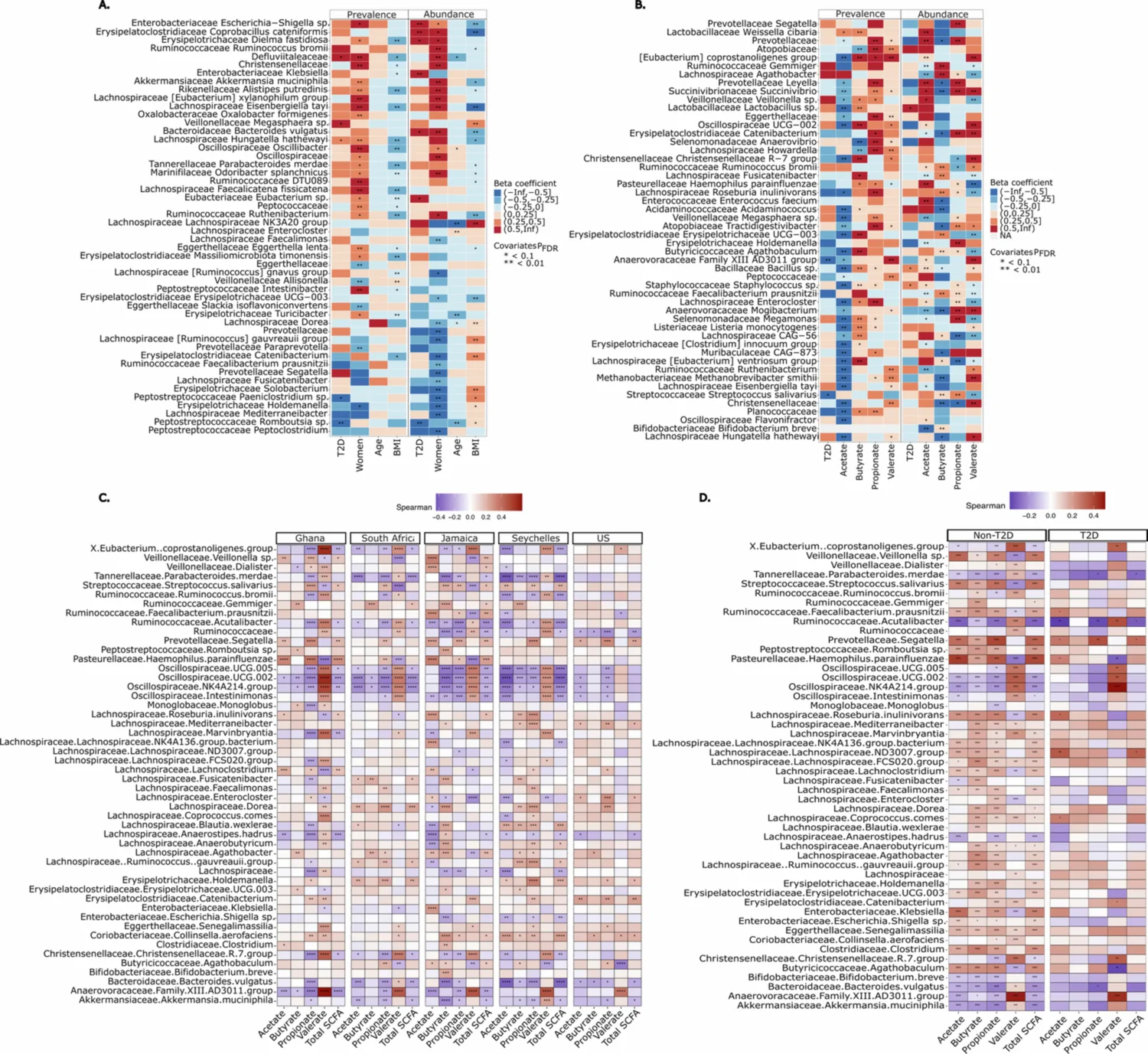
Differentially abundant taxa. (A) Top 50 significant taxa associated with T2D status by the model in MaAsLin3 (*n* = 1505). (B) Top 50 significant taxa associated with variables in (A), plus SCFAs (*n* = 1446). For panel A–B, MaAsLin3 analyses were adjusted for site, sex, BMI, and age, significance was determined as the q-value and taxa in the heatmap were ordered by significance for T2D. Positive associations were designated with (+) and negative associations were designated with (−) in the heatmaps. (C) Spearman correlation coefficients between the significant taxa from (A) and (B) and SCFAs stratified by country of origin. (D) Spearman correlation coefficients between the significant taxa from (A) and (B) and SCFAs stratified by participants with T2D and without T2D (non-T2D). Reported correlation coefficients with *p*-value < 0.05 were indicated as: *p < 0.05; **p < 0.01; ***p < 0.001, ****p < 0.0001.

The significance of these correlations between the top T2D-associated taxa from Figure 2A and B and SCFAs varied by site (Figure 2C). The taxon abundance among the Ghanaians tended to be more strongly correlated with each of the SCFAs. On the other hand, there were fewer statistically significant correlations between taxa abundance and SCFAs in US participants. Across the sites, a number of the taxa with the strongest significant negative correlations with acetate, butyrate, propionate, and total SCFAs (including multiple taxa from the *Oscillospiraceae family* and *Family XIII AD3011 group*) were, in contrast, strongly positively associated with valerate (Figure 2C). While the direction, if not the significance, of most of the correlations remained similar between sites, although not for all taxa. For example, *Lachnospiraceae enterocloster* was positively correlated with acetate in Jamaica and the US, but negatively in the Seychelles.

Correlations of the top T2D-associated taxa with SCFAs by T2D are presented in Figure 2D. Overall, the direction of the correlations between taxa and SCFAs was similar for both T2D and non-T2D status. However, more taxa showed significant correlations with SCFAs in the non-T2D group compared to the T2D group. In the non-T2D group, taxa positively correlated with acetate, butyrate, propionate, and total SCFA, including *Prevotellaceae Segatella*, *Romboutsia,* and *Enterobacteriaceae Klebsiella*. The latter was negatively correlated with valerate. On the other hand, *Family XIII AD3011 group*, *Ruminococcaceae Acutalibacter*, and multiple taxa in the *Oscillospiraceae* family were negatively correlated with acetate, butyrate, propionate, and total SCFA (and positively with valerate) in the non-T2D group. The few significant results observed in the T2D group tended to mirror these findings, particularly for valerate.

### Predicted functional pathways that differed significantly by T2D status included SCFA-related metabolic pathways

Since our results highlighted differences in the gut microbiota composition by T2D status, we characterized the functional pathways of taxa by T2D. The average relative abundance of taxa associated with each metabolite in the KEGG pathways are presented in Figure 3. Using PICRUSt2 predicted KEGG functional pathways, and four metabolism pathway classes (carbohydrate metabolism, glycan biosynthesis and metabolism, metabolism of terpenoids and polyketides, and biosynthesis of other secondary metabolites), one environmental information processing class (signal transduction), and one organismal systems class (nervous systems) were different by T2D. Butanoate metabolism was observed to have a significant positive log-fold change in those with T2D compared to those without T2D.

**Figure 3.**
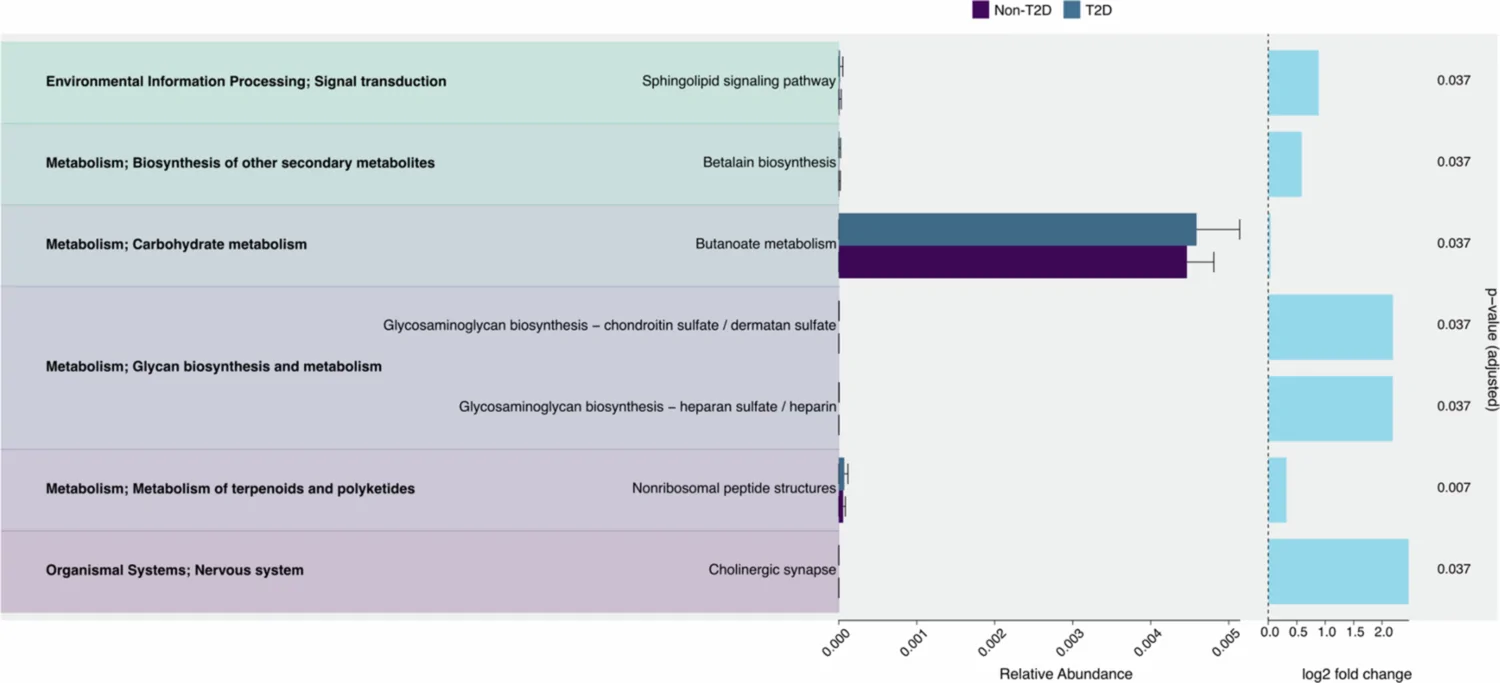
Significant taxa as top enriched functional pathways. The top taxa across all the sites were used as an input table for PICRUSt2 analysis. KEGG orthologs (KO) are annotated to KEGG pathways characterized for significant taxa across all sites. Pathway class, pathway name, relative abundance by site, log2 fold change in KOs by group status, and adj *p*-value are listed from left-to-right. The relative abundances are summed for all taxa with KOs along the labeled KEGG pathway, representing a community summed relative abundance measure for each identified top pathway.

### Taxa, SCFAs, and clinical measures predict current T2D status

To understand the contribution of the microbiome and SCFAs to the classification of current T2D status relative to clinical features (including glucose metabolism parameters, risk factors, and comorbidities), these variables were included in the machine learning predictive models (Figure 4A and B). The microbial taxa outperformed some clinical measures including, with 19 taxa ranking among the top 30 predictive features in the predictive model. The eight taxa with the greatest contribution to model output included *Desulfovibrionaceae Desulfovibrio, Enterobacteriaceae Klebsiella, Lachnospiraceae CAG−56, Eubacterium sp., Synergistaceae Cloacibacillus porcorum, Lactobacillus sp., Spirochaetaceae Treponema succinifaciens,* and *Escherichia−Shigella sp.* Among the SCFAs, valerate ranked among the top 30 features contributing to model output (Figure 4B). A variation of the machine learning model that included only the microbiota taxa, without any clinical features (Supplementary Figure 3), showed weak discriminatory performance for current T2D status, with an average bootstrapped AUC of 0.69.

**Figure 4.**
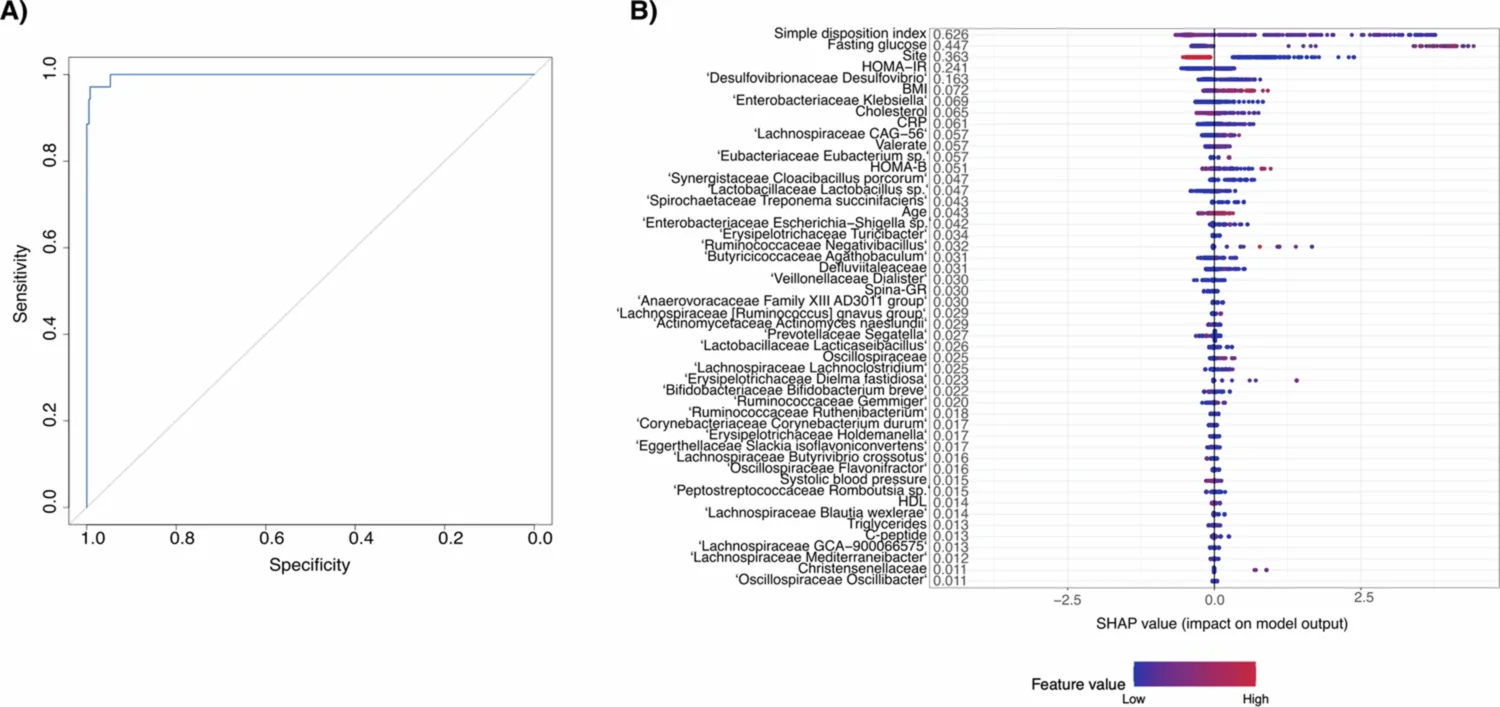
Taxa, SCFAs, and clinical metadata predict T2D status in all sites combined. Clinical metadata were the most predictive features of T2D, but some identified taxa were more strongly predictive than some clinical variables, ranking in the top 30 predictor variables. Model outcome (T2D) was more strongly associated with selected taxa than with some clinical variables. Valerate ranked among the top 15 predictors in the model.

## Discussion

Building upon an existing cohort of African origin adults from five countries with varying geographic and economic characteristics, we explored cross-sectional relationships between the gut microbiota, fecal SCFAs, T2D status, and markers of glucose metabolism. Our results indicate significant variations in gut microbiota diversity and composition between the research sites and by T2D status, with microbial diversity also associated with secondary glucose metabolism markers and several taxa differentially associated with T2D. We confirmed previous findings that participants’ geographic setting accounts for substantial variation in gut microbial composition and diversity, [28,48] with both microbial diversity and fecal SCFA levels decreasing with increasing site urbanization and development index (Ghana to US). Several unmeasured variables across sites, including site-related differences in diet, medication use, and climate impacts, etc., may partly explain these associations. However, several microbial taxa were associated with T2D after adjusting for site. Notably, we found that microbial taxa that were strongly associated with T2D status were often significantly correlated with fecal SCFAs across sites, although these associations differed by site. In terms of functional pathways, the predicted butanoate metabolic pathway was found to differ by T2D status, being enriched in participants with T2D. Taken together, these findings provide further insight into the complex relationship between T2D, the gut microbiota, and fecal SCFAs, which may differ by geographic setting.

The prevalence of T2D was found to vary from 2% in South Africa, 3% in Ghana, to 20% among the US participants. In the US site, participants are from a predominantly non-Hispanic Black community, where the T2D prevalence, in part, reflects the disproportionately higher impact of T2D, obesity, and other cardiometabolic parameters among non-Hispanic Black populations in the US [49,50]. In addition, the prevalence trends of T2D and impaired glucose metabolism across countries' economic development are largely in line with epidemiologic and nutritional transitions, reflecting differences in obesity prevalence, population lifestyles, cultural norms, socio-structural factors, and dietary shifts from fiber-rich, traditional diets to ultra-processed, high-calorie foods [51–53]. In fact, previous METS analyses have confirmed, based on 24-h recall data from 2010 from all sites except South Africa, that the US participants had lower total fiber intake (14.2 ± 7.1 g) compared to the other sites, with Ghana having the highest fiber intake (24.9 ± 9.7 g) [54]. Similarly, more recently (2021), as yet unpublished, 24-h dietary recall data from our METS study indicates that the median total fiber intake decreased from 20.1g (95%CI 14.5–26.9 g) in Ghana to 19.2 g (14.2–25.4 g) in Jamaica, 17.3 g (13.0–20.7 g) in South Africa, 15.8 g (11.4–21.6 g) in Seychelles, and finally 15.2 g (11.5–19.7 g) in the US, illustrating the varying degrees of the shift in global dietary patterns towards low-fiber and ultra-processed foods.

Our current results highlight a corresponding reduction in both gut microbiota alpha diversity and fecal SCFA levels by site, supporting the concept that the loss of dietary fiber negatively impacts SCFA production through the gut microbiota [55–57]. Interestingly, despite a lower diabetes prevalence (2%), the overall SCFA levels in South African participants were similar to those in participants from the US site. This may reflect a highly urbanized, socioeconomically disadvantaged group of South Africans, with a high intake of ultra-processed foods, [58–60] with the lower T2D prevalence, possibly partly explained by the younger median participant age (35 y) at this site. Together, these findings may signal that the future metabolic risk in this South African setting could rival the US site, warranting further investigation.

Several studies have reported lower alpha diversity among individuals with T2D compared with those without T2D [61–63]. Although we found statistically significant differences between T2D groups when pooling the sites for Shannon index and Chao1, these differences were not significant in analyses stratified by individual sites. Although the smaller within-site sample size may have impacted power to detect significant differences, particularly among sites with fewer cases of T2D, regression analysis adjusted for site, sex, age, and BMI also showed no significant association between T2D status and Shannon alpha diversity (Figure 1C). This suggests that the variation in alpha diversity may, in fact, reflect differences according to obesity and site (and unmeasured site-related factors, including diet) [64]. In addition, for the more urbanized, higher development index sites and lower alpha diversity, within-site variations caused by T2D status may be masked by a general decrease in alpha diversity due to the pervasiveness of ultra-processed foods, obesity, and other environmental factors [28,65,66].

We identified a negative association between *Romboutsia sp.* and T2D status. These findings support existing evidence that T2D risk and hyperglyc emia are associated with lower abundance of *Romboutsia* [61,67,68]. The causal direction and potential mechanisms by which this taxa may impact T2D pathophysiology remain unclear. Although existing studies have been conducted among participants of European descent, a study including African origin Surinamese participants similarly found a negative association between *Romboutsia* and metformin-treated T2D, although not among participants with treatment-naïve T2D [67]. The impact of T2D treatment in our setting requires further clarification. Of note*, Romboutsia,* which has previously been identified as an SCFA-producer, [19,61] was also positively correlated with fecal SCFA levels (particularly butyrate) in our study, but only in the non-T2D group. We hypothesize that this may be due to lower overall fecal SCFA levels and to the smaller sample size in the T2D group, although we were unable to explore the role of T2D treatment/metformin use (and between-site differences in medication use) in these associations. Additional research could help clarify these relationships and whether other T2D-related factors influence the production of SCFAs.

Several differentially abundant taxa positively associated with T2D, including taxa not widely identified in previous gut microbiota-T2D studies, such as *Dielma fastidiosa* [69] or *Coprobacillus cateniformis,* [70,71] as well as those previously linked to T2D risk, including *Escherichia−Shigella sp.* and *Bacteroides vulgatus* [72–76]. *Escherichia−Shigella* has also been associated with lipopolysaccharide production, increased gut permeability, and resulting inflammation [72] Although the evidence is inconclusive, a limited number of studies have also found that *Bacteroides vulgatus* is associated with metabolic risk factors, including obesity and insulin resistance [73–76]. Our results align with findings from 277 Danish participants, which identified *Bacteroides vulgatus* as a major taxon driving associations between the serum metabolome and insulin resistance [76].

Current evidence suggests that SCFAs may impact T2D risk through multiple mechanisms, including the stimulation of gut hormones such as glucagon-like peptide 1 (GLP-1), driving intestinal gluconeogenesis, eliciting direct peripheral effects e.g. on adipose tissue, and impacting pancreatic β-cell function and glucose-stimulated insulin secretion [11,14]. The first two of these mechanisms seem to be driven primarily by butyrate and propionate. Our results identified enrichment of butanoate metabolism in those with T2D, although this may represent increased turnover, compensatory pathways, or functional shifts, rather than increased butyrate production, particularly since we did not find evidence for higher butyrate concentrations in the T2D group (Supplementary Table 1). The functional pathway analysis may also reflect differences in site or BMI.

While the strong correlations between taxa differentially abundant by T2D status and fecal SCFA levels support existing evidence of a relationship between the gut microbiota, SCFAs, and T2D risk, [77] differences in these associations between sites suggest that other factors, including dietary intake, environmental, and overall SCFA concentrations, may play a key modulatory role [48,78–80]. Notably, more statistically significant associations were identified at the Ghanaian site, characterized by higher fiber intake, alpha diversity, and fecal SCFA levels, compared to the US, possibly reflecting a microbial community less favorable to SCFA production. The direction of associations between microbial taxa and valerate was often opposed to that of the three main fiber-fermented SCFAs (acetate, butyrate, propionate). This may reflect valerate's distinct production pathways, via amino acid fermentation or the chain elongation of shorter molecules (including SCFAs, [81]), suggesting that the taxa that shift in abundance with T2D status (e.g., *the Lachnospiraceae* family [82]) span these multiple metabolic processes. Interestingly, valerate was highest at the USA site, which also had the highest T2D prevalence, and was identified as a feature contributing to the predictive model’s classification of current T2D status (Figure 4). However, the implications of these patterns for metabolic risk remain largely unexplored.

Although the direction, if not the significance or strength of most SCFA associations, was similar between sites, this was not the case for all the taxa, including, for example, *Lachnospiraceae NK4A136 group* and *Lachnospiraceae enterocloster.* The site-dependence of these associations implies the potential impact of a broader bacterial network on SCFA enrichment over and above the species' own SCFA production. This provides insight into the complex interplay within gut microbial communities, which, although not yet well understood, may provide meaningful functional understanding beyond individual taxa or dichotomous enterotypes [83,84]. Further research is needed to clarify the mechanisms driving these associations and interactions in different settings and to explore whether targeting the gut microbiota composition and SCFA production could offer novel strategies for T2D prevention and control.

## Strengths and limitations

Our study has many notable strengths, including the large sample size, representing a diverse cohort of African origin adults with deep phenotyping allowing for adjustment for individual covariates as well as the exploration of site-level effects. The microbiome analysis includes standardized statistical methods for assessing diversity, differential abundance, and metabolic functional pathways, including the use of a state-of-the-art predictive machine learning model with a gradient boosting machine. However, the study should also be interpreted in the context of its limitations. One such limitation is the cross-sectional design, limiting our ability to make causal interpretations. In addition, the classification of T2D was based on a single fasting glucose measurement, as opposed to an oral glucose tolerance test (OGTT), which may be more sensitive in Black African populations [85–88]. Consequently, this study may have underestimated the prevalence of T2D. In addition, the statistical power of the study was lower because of the relatively low number of participants with T2D, particularly in individual site analyses (from *n* = 6 participants with T2D at the Ghanaian site to *n* = 43 at the US site). As a result of this lower power, the analysis also did not distinguish further between untreated and treated T2D. Despite this, however, we found significant associations among the gut microbiota, SCFAs, and glucose metabolism. Future studies with larger samples could conduct formal interaction analysis to determine whether T2D-microbiome composition and diversity associations differ by site. Although we adjusted for several possible confounders and relevant covariates, residual confounding may have resulted due to lack of adjustment for certain covariates (including medication use and dietary intake). As such, we were unable to draw definitive conclusions about the direct associations of fiber intake with the gut microbiome, SCFAs, and T2D, as fiber intake analysis was outside the scope of the current analysis. However, future research within the METS-Microbiome cohort leveraging detailed dietary data will further elucidate these associations, with the potential to provide novel insights into the role of dietary fiber in the composition of the gut microbiota and the production of SCFAs in various settings. We used 16S rRNA sequencing, which has limited species-level resolution, particularly for closely related taxa. Lastly, our study used fecal SCFA concentrations, which may not precisely reflect the levels of circulating SCFAs and are not a direct measure of SCFA production due to intestinal absorption and metabolism; future analyses including measures of both circulating and fecal SCFAs may provide further insights. Future research is needed to validate our findings in different contexts and explore causal mechanisms.

## Conclusion

Despite most of the microbiome variation identified in this cross-sectional analysis likely being attributable to developmental, dietary and other ecological site differences, we identified differentially abundant taxa by T2D status across the combined participant sample. The correlations between these T2D-associated taxa and SCFAs, which varied by site, nonetheless support the role of SCFAs in glucose metabolism. This paves the way for further investigation into the relationships between SCFAs, T2D risk, and population lifestyles, particularly within the context of shifting food environment and dietary (fiber) patterns in countries undergoing rapid nutritional and epidemiologic transitions. As such, microbiota-targeted interventions, including those addressing environmental or structural determinants of the microbiota and its metabolic products, may require adaptation to local environmental, population, and ecological contexts.

## Supplementary Material

Supplementary material STROBE checklist_T2DMicrob.docx

Supplementary material Supplementary Files_Table 1_Figures 1 2 3_25082026.docx

## Data Availability

All 16 S rRNA gene sequence data have been deposited at the European Bioinformatics Institute site under the accession code (https://www.ebi.ac.uk/ena/browser/view/PRJEB63378). Additionally, sequencing data and processed tables are available through QIITA under study identifier 13512. The SILVA 16 S rRNA database used for alignment is available at^117^ (https://data.qiime2.org/2022.2/common/silva-138-99-515-806-nb-classifier.qza). In line with privacy regulations of our cohort, the clinical and metadata are available upon reasonable request from LRD (https://lara.dugas@uct.ac.za).

## Abbreviations

ASV: amplicon sequence variants
BMI: body mass index
hsCRP: high-sensitivity C-reactive protein
HDI: human development index
HDL: high-density lipoprotein
HOMA-IR: homeostatic model assessment of insulin resistance
HOMA-β: homeostasis model assessment of beta-cell function
IQR: interquartile range
KEGG: kyoto encyclopedia of genes and genomes
LDL: low-density lipoprotein
LMIC: low- and middle-income country
MaasLin3: microbiome multivariate association with linear models 3
METS: modeling the epidemiologic transition study
OGTT: oral glucose tolerance test
PERMANOVA: permutational analysis of variance
PICRUSt: phylogenetic investigation of communities by reconstruction of unobserved states 2
SCFA: short chain fatty acids
T2D: type 2 diabetes
US: United States
