# Supplementary material for: Type 2 diabetes-associated gut microbial composition and short chain fatty acids differ across the epidemiologic transition in African origin populations
Source: Gut Microbes Rep. 2026 Oct 7;4(1):2741991. doi: 10.1080/29933935.2026.2741991 (PMC13652081; doi:10.1080/29933935.2026.2741991)
Supplement: Supplementary file 1 [file KGMR_A_2741991_SM7725.docx]

**Supplementary Table 1**: significance testing of SCFAs by T2D status

| SCFA | T2D | No T2D | P-value |
| --- | --- | --- | --- |
| Acetate | 2105.1 (1289.4-3097.5) | 2199.5 (1298.4-3840) | 0.234 |
| Propionate | 968.4 (665.2-1534.9) | 1119.4 (702.7-1800) | 0.180 |
| Butyrate | 741.7 (480.2-1030.7) | 796.0 (483.0-1300.1) | 0.293 |
| Valerate | 107.3 (61.0-180.4) | 105.0 (57.1-164.0) | 0.434 |
| Total SCFA | 4318.2 (2936.5-5396.6) | 4519.8 (2898.6-6907.4) | 0.144 |

Values are Median, IQR; Using Mann-Whitney U test

**Supplementary Figure 1: Alpha diversity is not statistically significantly different by T2D status within each site.** A) Shannon and B) Chao1 Alpha diversity by T2D status within each individual site (p>0.05 for difference between groups for each site).

**Supplementary Figure 2: Top 50 significant taxa associated with site in the MaAsLin3 model,** adjusted for sex, BMI, age, and T2D (n=1505), with Ghana as the reference site.

**Supplementary Figure 3: Taxa metadata alone are weakly predictive of T2D status in all sites combined.** Model outcome (T2D) was associated with select taxa; the average bootstrapped area under the curve (AUC) for the predictive model was 0.69.
